# Supplementary material for: Overexpressing eukaryotic elongation factor 1 alpha (eEF1A) proteins to promote corticospinal axon repair after injury
Source: Cell Death Discov. 2022 Sep 20;8:390. doi: 10.1038/s41420-022-01186-z (PMC9485247; doi:10.1038/s41420-022-01186-z)
Supplement: Supplementary file 1 — Supplementary Figures [file 41420_2022_1186_MOESM1_ESM.pdf]

Supplementary information

Overexpressing eukaryotic elongation factor 1 alpha (eEF1A) proteins to promote corticospinal axon repair after injury

Daniel Romaus-Sanjurjo, Junmi M. Saikia, Hugo J. Kim, Kristen M. Tsai, Geneva Q. Le, and Binhai Zheng

Supplementary figures with legends

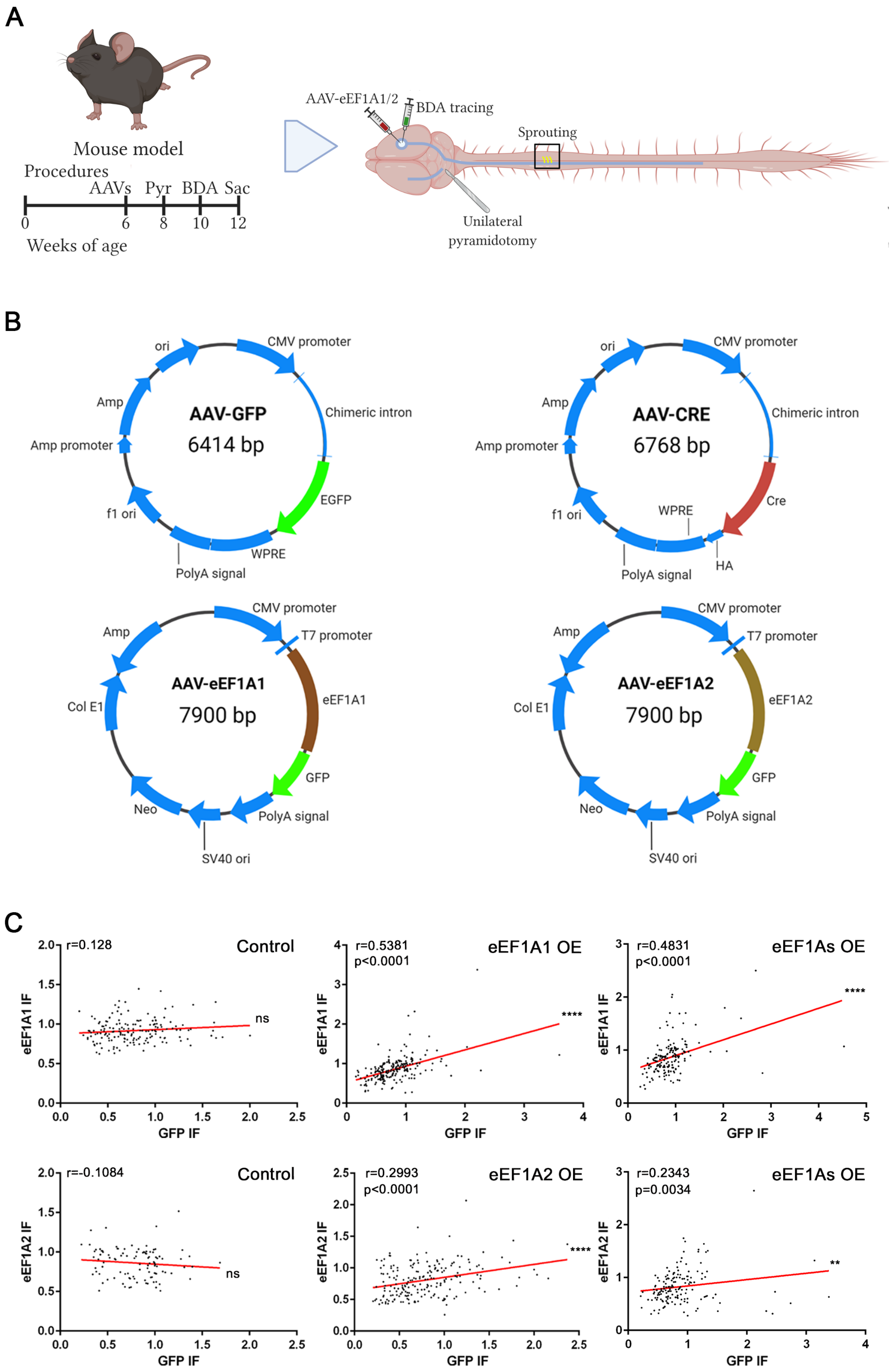
A
Mouse model
Procedures
AAVs Pyr BDA Sac
0 6 8 10 12
Weeks of age
AAV-eEF1A1/2
BDA tracing
Sprouting
Unilateral pyramidotomy
B
AAV-GFP
6414 bp
ori
CMV promoter
Chimeric intron
EGFP
WPRE
PolyA signal
f1 ori
Amp promoter
Amp
AAV-CRE
6768 bp
Cre
HA
AAV-eEF1A1
7900 bp
T7 promoter
eEF1A1
GFP
Neo
SV40 ori
Col E1
AAV-eEF1A2
7900 bp
eEF1A2
C
Control
r=0.128
ns
eEF1A1 OE
r=0.5381
p<0.0001
eEF1As OE
r=0.4831
p<0.0001
eEF1A1 IF
GFP IF
Control
r=-0.1084
eEF1A2 OE
r=0.2993
p<0.0001
r=0.2343
p=0.0034
eEF1A2 IF

Fig. S1. Experimental paradigm, viral vectors, and correlation analyses on eEF1A1 and eEF1A2 overexpression. A) Experimental timeline and injury model illustration. B) Different viral constructs used in this study. C) Positive correlation for eEF1A1 or eEF1A2 immunofluorescence (IF) signals with GFP tag IF signals in eEF1A1 OE, eEF1A2 OE and eEF1As OE mice. Control, 12 mice; eEF1A1, 10 mice; eEF1A2, 10 mice; eEF1As, 8 mice; 20 cells quantified per mouse. Stats: Spearman correlation coefficient test (eEF1A1 IF: control, $r = 0.128$, $p = 0.0981$; eEF1A1 OE, $r = 0.5381$, $p < 0.0001$; eEF1As OE, $r = 0.4831$, $p < 0.0001$. eEF1A2 IF: eEF1A2 OE, $r = 0.2993$, $p < 0.0001$; eEF1As OE, $r = 0.2343$, $p = 0.0034$); and Pearson correlation coefficient test (eEF1A2 IF: control, $r = -0.1084$, $p = 0.2712$).

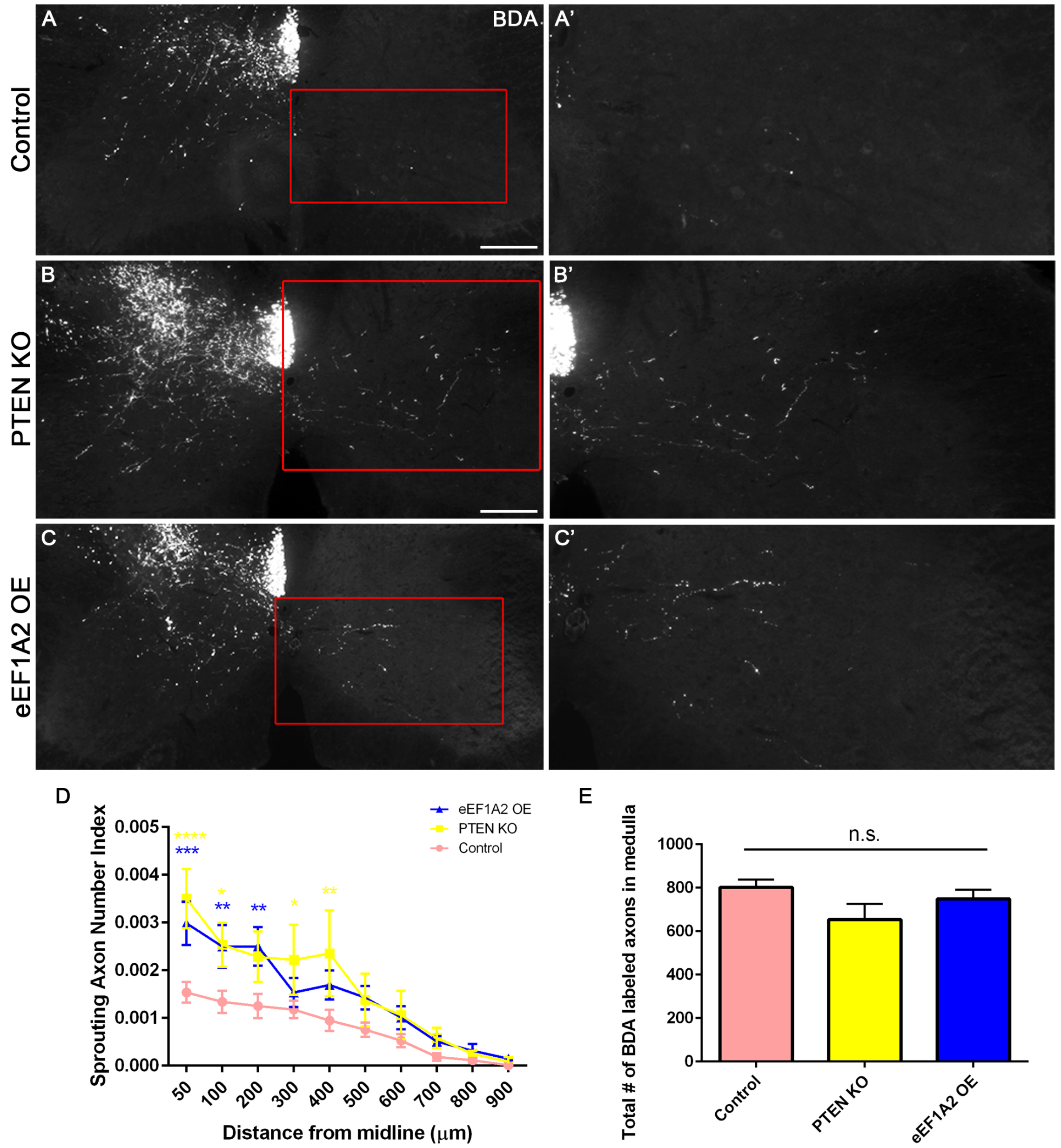

Fig. S2. A comparison of CST sprouting phenotype between eEF1A2 overexpression mice and PTEN deletion mice. A-C') Representative images of BDA tracing at the level of cervical spinal cord in control (A-A'), PTEN KO (B-B'), and eEF1A2 OE (C-C') mice. Scale bar = 300 μm. Right panel shows individual sprouting axons in the grey matter of the denervated half of spinal cord. D) Quantification of sprouting index in control, PTEN KO and eEF1A2 OE mice. Compared to control, two-way RM ANOVA multiple comparisons with Tukey's correction revealed elevated sprouting for PTEN cKO: at 50 μm, $p < 0.0001$; at 100 μm, $p = 0.0263$; and at 300 μm, $p = 0.0482$; and at 400 μm, $p = 0.007$. Elevated sprouting for eEF1A2 OE compared to controls was observed at 50 μm, $p = 0.0001$; at 100 μm, $p = 0.0031$; and at 200 μm, $p = 0.0013$. Note that eEF1A2 OE mice exhibit compensatory CST sprouting at levels similar to PTEN KO mice. E) Quantification of BDA-labeled axons at medullas. One-way ANOVA revealed no significant differences. Bars show mean ± SEM. Control mice: n = 12; eEF1A2 OE mice: n = 9; and PTEN cKO mice: n = 4.

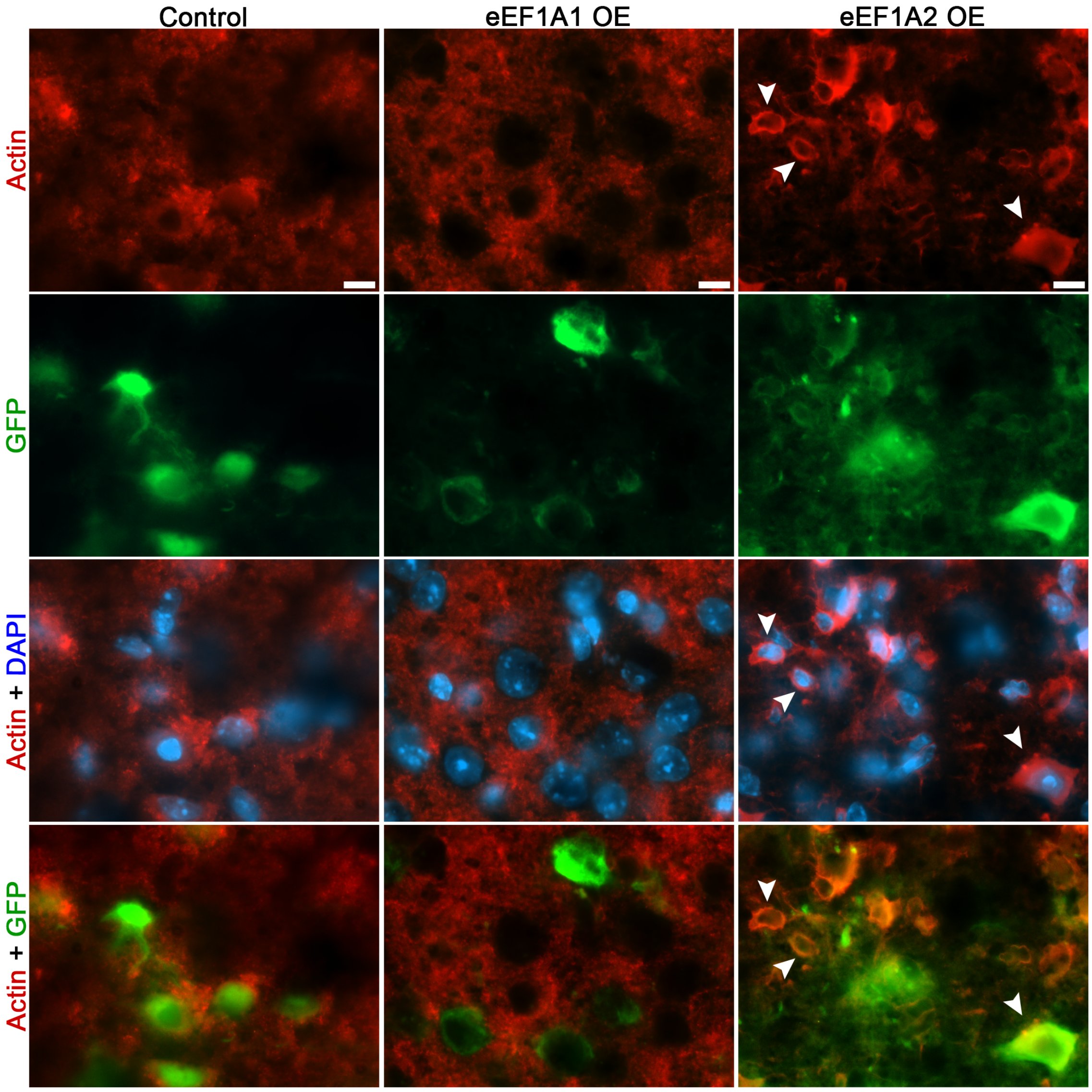

Fig. S3. High magnification images of actin bundling in eEF1A2 overexpression mice. Representative 100× images of β-actin and GFP (viruses) staining in the right sensorimotor cortex of control, eEF1A1 OE, and eEF1A2 OE mice. Note the bundled pattern of β-actin staining defining the cellular shape in neurons GFP^{+} from eEF1A2 OE mice (arrowheads). Scale bars = 10 µm.
